# Associations between immuno-metabolic markers, working memory, and functional brain activation in depressive and anxiety disorders

**DOI:** 10.1016/j.bbih.2026.101369

**Published:** 2026-09-22

**Authors:** Simon Braak, Laura K.M. Han, Chris Vriend, Thomaz F.S. Bastiaanssen, Brenda W.J.H. Penninx

**Affiliations:** a Amsterdam UMC, Location Vrije Universiteit Amsterdam, Department of Psychiatry, De Boelelaan 1117, Amsterdam, the Netherlands; b Amsterdam Neuroscience, Mood, Anxiety, Psychosis, Sleep & Stress and Neurodegeneration Programs, Amsterdam, the Netherlands; c Amsterdam Public Health, Mental Health Program, Amsterdam, the Netherlands; d Amsterdam UMC, Location Vrije Universiteit Amsterdam, Anatomy and Neurosciences, De Boelelaan 1117, Amsterdam, the Netherlands; e Amsterdam Neuroscience, Brain Imaging, Amsterdam, the Netherlands

**Keywords:** Inflammation, Metabolic syndrome, Metabolomics, Working memory, N-back task, Functional activation, Depression, Anxiety

## Abstract

**Background:**

Cognitive impairment and immuno-metabolic dysregulation are commonly observed in depressive and anxiety disorders, but underlying brain processes remain unclear. This study examined relationships between immuno-metabolic markers, working memory performance, and task-related functional brain activation in persons with depressive and anxiety disorders and healthy controls (HC).

**Methods:**

Functional MRI data acquired during an n-back task were analyzed in 54 persons with depressive and anxiety disorders and 69 HC to assess working memory load-related activation. Working memory performance was indexed using d-prime and reaction time. Peripheral immuno-metabolic characteristics were assessed using an inflammation composite index (c-reactive protein, interleukin-6, tumor necrosis factor-alpha, interferon-gamma), a metabolic syndrome index, and a principal component analysis-derived metabolomics factor based on 72 circulating metabolites.

**Results:**

Across all participants, the working memory task significantly induced functional activation in canonical task-related brain regions (Z-max = 3.72, *p* < 0.05), although independent of test performance (*p > *0.05). No significant associations were found between immuno-metabolic measures and either working memory performance or functional activation, nor were any interaction effects with diagnostic status observed (*p > *0.05).

**Conclusions:**

We found no evidence to support a link between peripheral immuno-metabolic markers and working memory performance or related functional brain activation in persons with depressive and anxiety disorders and HC. Longitudinal and interventional studies incorporating more direct assessments of neuroinflammation will be necessary to further clarify the role of immuno-metabolic factors in cognition.

## Highlights

• Immuno-metabolic associations with cognition in depression and anxiety are unclear.
• The n-back fMRI task elicited expected functional brain activation across the sample.
• Immuno-metabolic markers did not relate to working memory or brain function.

## 1 Introduction

Cognitive impairment is commonly observed in depressive and anxiety disorders, leading to reduced quality of life and increased functional disability (Knight and Baune, 2018; Saragoussi et al., 2018). Yet, it is rarely a primary focus of routine clinical management, and few pharmacological treatment options specifically targeted at cognitive impairment are available (Perini et al., 2019). Emerging evidence has indicated that altered immune (e.g., elevated peripheral levels of C-reactive protein (CRP) and pro-inflammatory cytokines) and metabolic states (e.g., insulin resistance and altered lipid profiles) in depressive and anxiety disorders might be linked to cognitive impairment (Penninx et al., 2025; Sanchez-Carro et al., 2022; Maksyutynska et al., 2024; Morrens et al., 2022; Mehdi et al., 2024; Mac Giollabhui et al., 2025). While findings linking peripheral inflammation to global cognitive function in affective disorders have been weak, metabolic dysregulation has been more strongly linked to cognitive impairment, particularly working memory deficits (Maksyutynska et al., 2024; Morrens et al., 2022; Mac Giollabhui et al., 2025).

Immuno-metabolic dysregulation may impair cognitive function by modulating activity in brain regions critical for working memory and executive control (Penninx et al., 2025; Miller, 2025; Wang et al., 2019; Yaple et al., 2019; Joyce et al., 2025). Peripheral inflammatory signals are thought to reflect a proxy for neuroinflammatory mechanisms that may disrupt neurotransmission and thereby reduce the brain's capacity to respond efficiently to increased cognitive load (Joyce et al., 2025; Yirmiya and Goshen, 2011; Miller et al., 2013), while metabolic dysregulation, such as insulin resistance, may lead to decreased glucose uptake and subsequent signaling deficits in the brain (Malin et al., 2022; Williams et al., 2019). The n-back task is a widely used paradigm for assessing working memory capacity and, when performed inside an MRI scanner, for examining brain activation in response to increasing cognitive load, making it well suited for probing working memory in relation to immuno-metabolic factors (Wang et al., 2019; Nikolin et al., 2021). Prior meta-analyses have shown that the n-back task reliably demonstrates brain activation in frontal, parietal, insular, and cerebellar cortices, as well as the thalamus and caudate nucleus, with evidence for age-related shifts in functional involvement (i.e., greater frontal engagement in younger adults and greater parietal engagement in older adults) (Wang et al., 2019; Yaple et al., 2019).

Importantly, elevated peripheral inflammation and/or metabolic dysregulation have been linked to altered functional activation within many of these brain regions (i.e., prefrontal, insular, and cerebellar cortices and the caudate and thalamus) using diverse study designs, including inflammatory manipulations and observational studies employing resting-state, emotional, and cognitive paradigms (Kraynak et al., 2018; Yao et al., 2021; Dev et al., 2017). Nevertheless, to our knowledge, no study has investigated these relationships in persons with depressive and anxiety disorders using a working memory task, which is a crucial step towards identifying novel biology-based personalized treatment targets for cognitive impairment (Kas et al., 2025).

Therefore, in the current study, we examined how immuno-metabolic markers are related to working memory performance and associated functional brain activation in persons with depressive and anxiety disorders and healthy controls (HC). We hypothesized that elevated markers for peripheral inflammation and metabolic dysregulation would be associated with poorer working memory performance and lower functional activation in working memory related brain regions across diagnostic groups (Maksyutynska et al., 2024; Kraynak et al., 2018; Yao et al., 2021; Dev et al., 2017).

## 2 Methods

### 2.1 Participants

Data were acquired from the MOod Treatment with Antidepressant or Running (MOTAR) study (Lever-van Milligen et al., 2019). This intervention study investigated the impact of antidepressant medication versus running therapy on somatic and mental health, recruiting 141 persons with depressive and/or anxiety disorders between 2012 and 2019 (Lever-van Milligen et al., 2019). Baseline n-back fMRI task data was collected from 56 of these participants and from 70 HC to facilitate case-control comparisons. The study was approved by the Medical Ethical Committee VU University Medical Centre, and all participants provided written informed consent.

Inclusion and exclusion criteria have been described in detail elsewhere (Lever-van Milligen et al., 2019). In short, participants, aged between 18 and 70 years, met criteria for either a current major depressive disorder and/or an anxiety disorder (generalized anxiety disorder, social anxiety disorder, panic disorder, or agoraphobia), or were HC without a lifetime DSM-IV diagnosis. Diagnoses were assessed using the DSM-IV Composite International Diagnostic Interview (Robins et al., 1988). Depression severity was assessed with the Inventory of Depressive Symptomatology (IDS) and anxiety severity was assessed with the Beck Anxiety Inventory (BAI) (Rush et al., 1996; Beck et al., 1988). Exclusion criteria included primary psychiatric diagnoses other than depressive or anxiety disorders, MRI contraindications, and current psychotropic medication use (with the exception of stable benzodiazepines use).

### 2.2 Inflammatory and metabolic markers

High-sensitivity CRP, interleukin-6 (IL-6), tumor necrosis factor-alpha (TNF-α), and interferon-gamma (IFN-γ) concentrations were measured in serum samples. CRP concentrations were quantified in duplicate using an in-house enzyme-linked immunosorbent assay (ELISA, CRPHS, Roche Diagnostics, Indianapolis, IN, USA) based on purified protein and polyclonal anti-CRP antibodies (Dako, Glostrup, Denmark), with a lower detection limit of 0.15 mg/l and a sensitivity of 0.3 mg/l. Serum concentrations of IL-6, TNF-α, and IFN-γ were measured using a multiplex chemiluminescent immunoassay (V-PLEX Human Proinflammatory Panel I, Meso Scale Discovery, LLC. Rockville, MD, USA) on the MESO QuickPlex SQ 120 platform. The panel's lower limits of detection were 0.05–0.09 pg/ml for IL-6, 0.01–0.13 pg/ml for TNF-α, and 0.21-0.62 pg/ml for IFN-γ. A composite inflammation index was calculated as the mean of log-transformed and standardized concentrations of CRP, IL-6, TNF-α, and IFN-γ (Vreijling et al., 2025), and is considered to better reflect systemic inflammation than individual markers alone.

Metabolic health was assessed using a metabolic syndrome index and a principal component analysis (PCA)-derived metabolomics factor, with higher values reflecting poorer metabolic health (Vreijling et al., 2025). The metabolic syndrome index was based on five components: fasting glucose, high-density lipoprotein (HDL) cholesterol, triglycerides, systolic blood pressure, and waist circumference (Lamers et al., 2020). Triglyceride values were log-transformed, and all components were standardized. HDL cholesterol scores were reversed-scored after standardization, as lower levels reflect higher metabolic risk. Finally, the index was calculated by averaging the five standardized variables. Additionally, in line with Vreijling et al. (2025), we conducted a PCA on 72 metabolites (lipids, fatty acids, and low-molecular weight metabolites) quantified using a proton Nuclear Magnetic Resonance platform (Nightingale Healthy Ltd., Helsinki, Finland). A detailed description of the platform is provided in Bot et al. (2020). One metabolite from the originally planned set of 73 was excluded due to missing values (N = 8). Raw metabolite values were increased by 1, log-transformed, standardized, and truncated at ±5 standard deviations from the mean (Vreijling et al., 2025). The resulting first principal component has previously been shown to correlate with the inflammatory and metabolic syndrome index and to track changes in metabolic syndrome over time and was therefore used in subsequent analyses (Vreijling et al., 2025). This principal component accounted for 42.3% of the variance in metabolites and reflected a lipid-dominant profile, with positive loadings for very low-density and low-density lipoproteins (see Supplemental Table 1 for the metabolite loadings).

### 2.3 N-back fMRI task

A visual, letter-based n-back fMRI paradigm was used to assess working memory performance and related functional brain activation. The task included four load conditions (0-back, 1-back, 2-back, and 3-back), consisting of 16 blocks with 14 stimuli per block. Block order was fixed across participants. Each stimulus was presented for approximately 2.0 s, and an instruction screen preceded each block. In the 0-back condition, participants were instructed to press a button whenever the letter “X” was presented on the screen. In the 1-, 2-, and 3-back conditions, participants were instructed to press a button when the current letter matched the one presented 1, 2, or 3 trials earlier, respectively. The total task duration was approximately 9.5 min. Task performance was quantified with two primary outcomes: 1) d-prime (*d’)*, which provides an unbiased measure of accuracy (calculated as the difference between the standardized hit and false alarm rates) (Haatveit et al., 2010), and 2) reaction time on correct hits, calculated across the 1-, 2-, and 3-back conditions. For completeness, overall accuracy, defined as the percentage of correct responses across the 1-, 2-, and 3-back conditions, was also used as a measure of task performance. Finally, d-prime, reaction time, and accuracy were examined separately for each load condition.

### 2.4 MRI data acquisition and preprocessing

Imaging data were acquired at the REC Spinoza centre in the Netherlands using a Philips (Best, the Netherlands) 3T MRI scanner equipped with a SENSE-32 head coil. MRI acquisition details are provided in the Supplement.

Structural MRI data were processed using the FreeSurfer 7.1.1 recon-all pipeline, which includes skull stripping, bias field correction, intensity normalization, and tissue segmentation (Fischl, 2012). Task-based fMRI data were preprocessed using fMRIPrep v23.2.3, and included skull stripping, motion correction, slice timing correction, and susceptibility distortion correction using the ‘fieldmap-less’ approach. Functional data was registered to the Montreal Neurological Institute (MNI) 152 Nonlinear Asymmetric template with 2-mm isotropic resolution. Subsequently, the functional images were spatially smoothed with an 8-mm full width at half maximum Gaussian kernel, and temporally filtered with a high-pass Gaussian filter at 0.01 Hz (Esteban et al., 2020). Framewise displacement (FD) was calculated, and those with a mean root mean squared FD > 1 mm or > 20 vol with > 1 mm volume-to-volume displacement were excluded due to excessive head motion (Power et al., 2014).

### 2.5 Statistical analyses

All hypotheses and analyses were preregistered at https://osf.io/5s6c7.

#### 2.5.1 Demographic and clinical data

Group differences in demographic, clinical, and biological data were analyzed in R (version 4.3.2). Dichotomous variables were compared using chi-square (χ2) tests. Continuous variables were analyzed using independent-samples t-tests, and when parametric assumptions were violated, Mann-Whitney U-tests were applied. Correlations between the immuno-metabolic measures were calculated using Pearson's correlations.

#### 2.5.2 Relations between immuno-metabolic markers and working memory performance

To examine relationships between the inflammatory and metabolic markers and working memory performance, we conducted linear regressions with working memory d-prime and reaction time as the dependent variables and the inflammatory index, metabolic syndrome index, and the PCA-derived metabolomics factor as the independent variables in separate univariable models, correcting for age, sex, and diagnostic status (i.e., depression, anxiety, comorbid depression and anxiety, HC). For models showing significant associations, sensitivity analyses were planned that included benzodiazepine use, smoking status, and body mass index (BMI) as additional covariates to assess the robustness of findings. Additionally, we tested for interaction effects between diagnostic status (i.e., depressive/anxiety disorders vs. HC) and the immuno-metabolic indices. Finally, we performed a sensitivity power analysis based on the final sample to determine the smallest effect size detectable with 80% power (α = 0.05).

#### 2.5.3 Relations between immuno-metabolic markers and functional brain activation

Subject-level general linear model (GLM) analyses were performed using FMRIB's Improved Linear Model with local autocorrelation correction in FSL/FEAT (Woolrich et al., 2001). Separate regressors for the four task conditions (0-back to 3-back) were modeled as boxcar functions convolved with a double-gamma hemodynamic response function. To mitigate motion artefacts, 24 head-motion parameters (6 rigid-body, their temporal derivatives, and all squared terms) obtained from the fMRIPrep output were used as nuisance regressors in the subject-level GLMs. To examine effects of working memory load on neural responses, two primary contrasts were tested: a categorical contrast comparing 0-back versus 1- to 3-back conditions with weights [−3, 1, 1, 1], and a linear contrast across the 0-back to 3-back conditions with weights [−1.5, −0.5, 0.5, 1.5].

Using the subject-level statistical maps, group-level GLM analyses were performed using FSL's Randomise permutation-testing tool with N = 10,000 permutations with automatic outlier-deweighting (Woolrich et al., 2004; Winkler et al., 2014). An a priori working-memory network mask was created based on robustly activated brain regions identified in an n-back meta-analysis (Wang et al., 2019). The mask included the bilateral middle frontal gyrus, superior frontal gyrus, precentral gyrus, inferior parietal lobe, precuneus, insula, thalamus, caudate, and cerebellar structures (see Fig. 1). To allow an inclusive assessment, regions identified as lateralized in the prior meta-analysis (Wang et al., 2019) (i.e., reported only in the left or right hemisphere) were represented bilaterally in the mask. Cerebral regions were defined using the Harvard-Oxford anatomical atlas with a 10% probability threshold. For cerebellar regions, we used the Talairach atlas, which provides the best representation of the structures described in the prior meta-analysis (Wang et al., 2019).

**Fig. 1:**
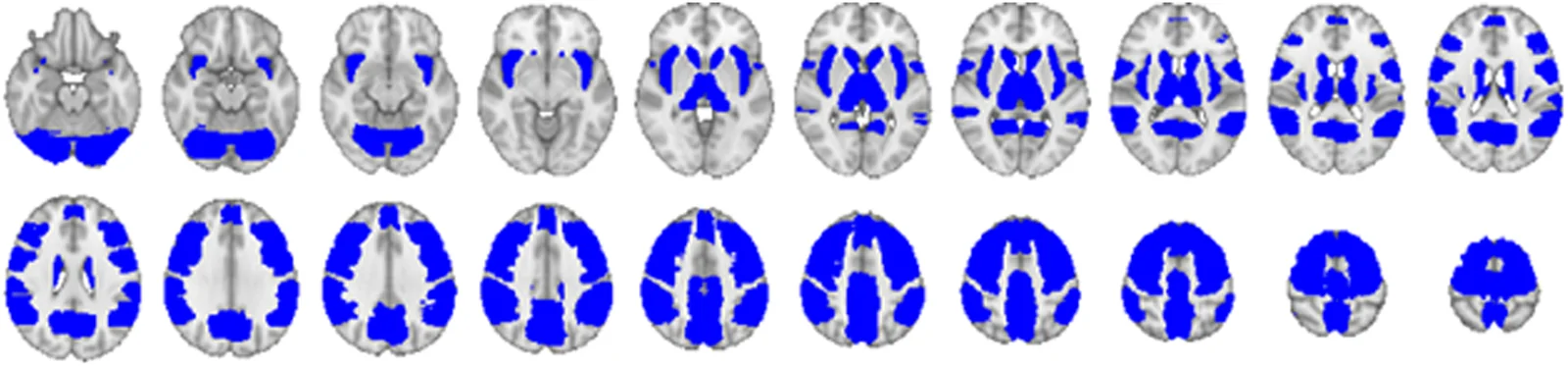
Axial view of the working-memory network pre-threshold mask. Image is displayed in radiological orientation, spanning from Montreal Neurological Institute coordinates Z = −20 (top left) to Z = 66 (bottom-right).

Case-control analyses were conducted to assess whether working memory load-related activation within the mask differed between groups. Additionally, separate linear regression models were used to test whether activation within the mask in response to working memory load is associated with: (1) the composite inflammation index, metabolic syndrome index, and PCA-derived metabolomics factor, and (2) working memory d-prime, reaction time, and accuracy (each tested in separate models) across the sample. All models were adjusted for age and sex. Diagnostic status (i.e., depression, anxiety, comorbid depression and anxiety, HC) was included as a covariate in the primary analyses of immuno-metabolic measures and in sensitivity analyses of working memory performance. If significant associations were observed, sensitivity analyses were planned to further adjust for benzodiazepine use, smoking status and BMI. Post-hoc analyses examined whether interaction effects between diagnostic status (i.e., depressive/anxiety disorders vs. HC) and either working memory performance or immuno-metabolic indices were associated with differences in working memory load-related functional activation. For completeness, analyses were also conducted at a whole-brain level. All independent variables were demeaned across groups prior to imaging analyses, and voxelwise maps were corrected using Threshold-Free Cluster Enhancement (TFCE) with family-wise error (FWE) correction at *p* < 0.05 (Smith and Nichols, 2009).

Finally, in addition to the preregistered analyses (https://osf.io/5s6c7), we conducted exploratory analyses to aid interpretation of the primary findings. Specifically, we examined whether symptom severity (i.e., IDS and BAI scores) was related to working memory performance and functional activation within the working memory mask across the sample, while controlling for age and sex. Moreover, we ran separate linear regression models to examine whether individual inflammatory markers (as opposed to the composite index) were related to working memory performance or functional activation within the working memory mask (Bonferroni-corrected for four tests, *p* < 0.0125). Finally, we repeated the functional activation analyses within the working memory mask using a categorical 0-back versus 2-back contrast with weights [−1, 0, 1, 0] and working memory performance measured in the 2-back condition. This contrast has previously been shown to be most sensitive to relations with working memory performance, and the 2-back condition has been reported to yield the largest effect sizes for accuracy impairments in persons with depression compared to HC (Nikolin et al., 2021; Owens et al., 2018). All voxelwise maps of the exploratory functional activation analyses were corrected using TFCE with FWE correction at *p* < 0.05 (Smith and Nichols, 2009).

## 3 Results

### 3.1 Participant and demographic variables

Clinical and demographic descriptive statistics are presented in Table 1. One HC participant was excluded due to excessive head motion and two persons with depressive and anxiety disorders did not have demographics/clinical data, leaving 123 participants for analyses (MDD: N = 11, ANX: N = 14, COM: N = 29, HC: N = 69). Given the final sample size (N = 123), a sensitivity power analysis indicated 80% power (α = 0.05) to detect an effect of Cohen's f^2^ = 0.066 in models examining associations between immuno-metabolic indices and working memory performance. In the majority of participants (82.6%), blood draw and MRI were conducted within two weeks (median: 7 days, IQR: 4-12), well within the period of strong to moderate temporal stability reported for CRP, IL-6, TNF-α, and IFN-γ (Walsh et al., 2023).

**Table 1:** Sample characteristics of each group.

	Persons with depressive and anxiety disorders (N = 54)	Healthy controls (N = 69)	Case-control differences
**Demographics**			

Age (years), mean (SD)	36.0 (11.6)	40.5 (14.5)	t(120.9) = −1.92, p = 0.057
Sex (% female)	57.4	46.4	χ2(1) = 1.48, p = 0.225

**Health factors**			

Smoking status (% current)	31.5	13.0	χ2(1) = 6.18, **p = 0.013**
Body mass index. mean (SD)	25.0 (4.3)	24.9 (4.4)	t(114.9) = 0.05, p = 0.960

**Psychotropic medication**			

Benzodiazepine use (%)	20.4	0.0	Odds ratio = 0.00, **p < 0.0001**

**Disorder severity**^a^			

Depression severity (IDS), mean (SD)	42.3 (14.2)	4.4 (3.8)	t(57.7) = 18.92, **p < 0.0001**
Anxiety severity (BAI), mean (SD)	24.7 (12.6)	1.9 (2.2)	t(54.5) = 13.01, **p < 0.0001**

**Working memory performance**			

d-prime, mean (SD)	3.4 (0.7)	3.6 (0.5)	t(96.5) = −1.75, p = 0.084
Reaction time, median (Q1 – Q3)	526.0 (475.9-604.0)	500.5 (432.1–562.3)	W = 2242, p = 0.054
Accuracy (%), median (Q1 – Q3)	87.5 (77.1–93.8)	91.7 (83.3–93.8)	W = 1547, p = 0.106

**Inflammatory**^a^			

Inflammation composite index, mean (SD)	0.1 (0.7)	−0.1 (0.7)	t(112.9) = 0.95, p = 0.346

**Metabolic**			

Metabolic syndrome index^b^, mean (SD)	0.0 (0.7)	−0.0 (0.8)	t(103.3) = 0.38, p = 0.708
PCA-derived metabolomics factor^c^, mean (SD)	−0.2 (5.2)	0.1 (5.7)	t(114.3) = −0.34, p = 0.732

a Sample size was N = 121.

b Sample size was N = 113.

c Sample size was N = 120. IDS = Inventory of Depressive Symptomatology. BAI = Beck Anxiety Inventory. PCA = principal component analysis.

Age and sex were comparable between depressive/anxiety disorders and HC (*p* > 0.05). As expected, symptom severity was significantly higher in persons with depressive and anxiety disorders compared to HC (*p < *0.0001). Eleven patients (20.4%) and none of the HC used benzodiazepines. Oxazepam was the most commonly used benzodiazepine (N = 8), with typical daily doses of approximately 30 mg, and only three participants used benzodiazepines for more than 6 months. The composite inflammation index was significantly but moderately correlated to the metabolic syndrome index (r = 0.45, *p < *0.0001) and the PCA-derived metabolomics factor (r = 0.35, *p < *0.0001), whereas the PCA-derived factor showed a strong correlation with the metabolic syndrome index (r = 0.64, *p* < 0.0001). Intercorrelations between inflammatory markers are provided in Supplemental Table 2. Working memory performance across the 1-, 2-, and 3-back conditions and the immuno-metabolic indices did not significantly differ between persons with depressive/anxiety disorders and HC (*p* > 0.05) (see Fig. 2), although marginal trends towards poorer performance in the patient group were observed (d-prime: t(96.5) = −1.75, p = 0.084; reaction time: W = 2242, p = 0.054). Additionally, across the sample, higher IDS and BAI scores were associated with lower d-prime (IDS: t(117) = −3.05, p = 0.003; BAI: t(117) = −2.76, p = 0.007) and accuracy scores (IDS: t(117) = −4.72, p < 0.0001; BAI: t(117) = −4.28, p < 0.0001). Similarly, higher IDS scores were associated with slower reaction times (t(117) = 2.85, *p* = 0.005), though this relationship was not significant for BAI scores (t(117) = 1.89, *p* > 0.05). For completeness, working memory performance metrics for each load condition are provided in Supplemental Table 3. Compared to HC, persons with depressive and anxiety disorders had lower d-prime scores and accuracy scores in the 1-back and 2-back conditions, and slower reaction times in the 1- and 3-back condition (all *p* < 0.05).

**Fig. 2:**
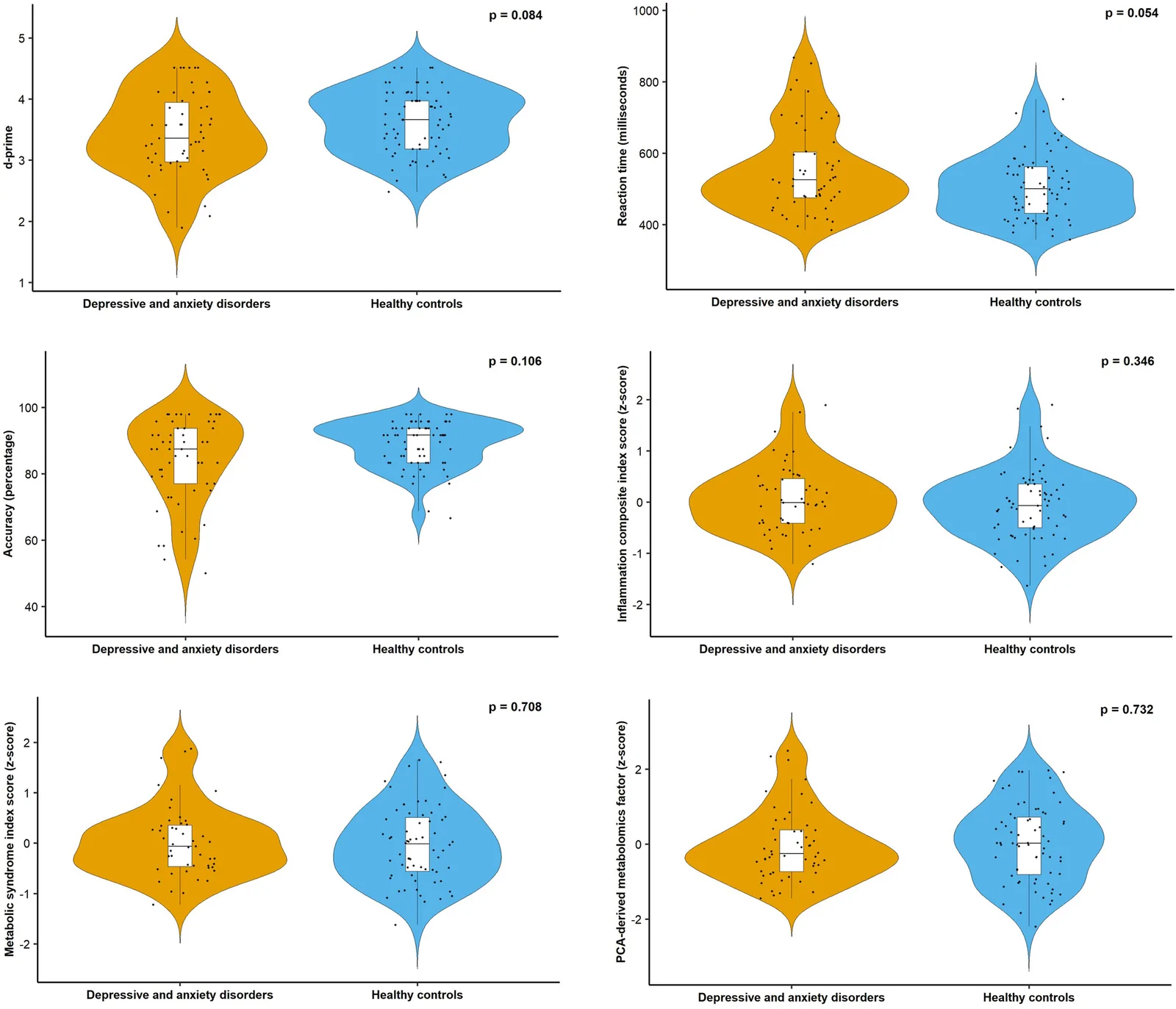
Group comparisons of working memory performance and immuno-metabolic markers. PCA = principal component analysis. Individual data points with horizontal jitter (for visibility purposes) are provided.

### 3.2 Relations between immuno-metabolic markers, working memory, and functional activation

All working memory analyses were conducted using both the categorical and linear load-related contrasts. Across all participants, the task significantly induced functional activation within large sections of the working memory mask (*p* < 0.05; Fig. 3), confirming that the task robustly elicited the expected activation. Whole-brain analyses revealed a similar pattern that was more spatially distributed (all *p* < 0.05); Supplemental Fig. 1). No differences between persons with depressive and anxiety disorders and HC were observed in functional activation within the working memory mask or across the whole-brain (*p* > 0.05). Additionally, IDS and BAI scores were not associated with functional activation within the working memory mask across the sample (*p* > 0.05).

**Fig. 3:**
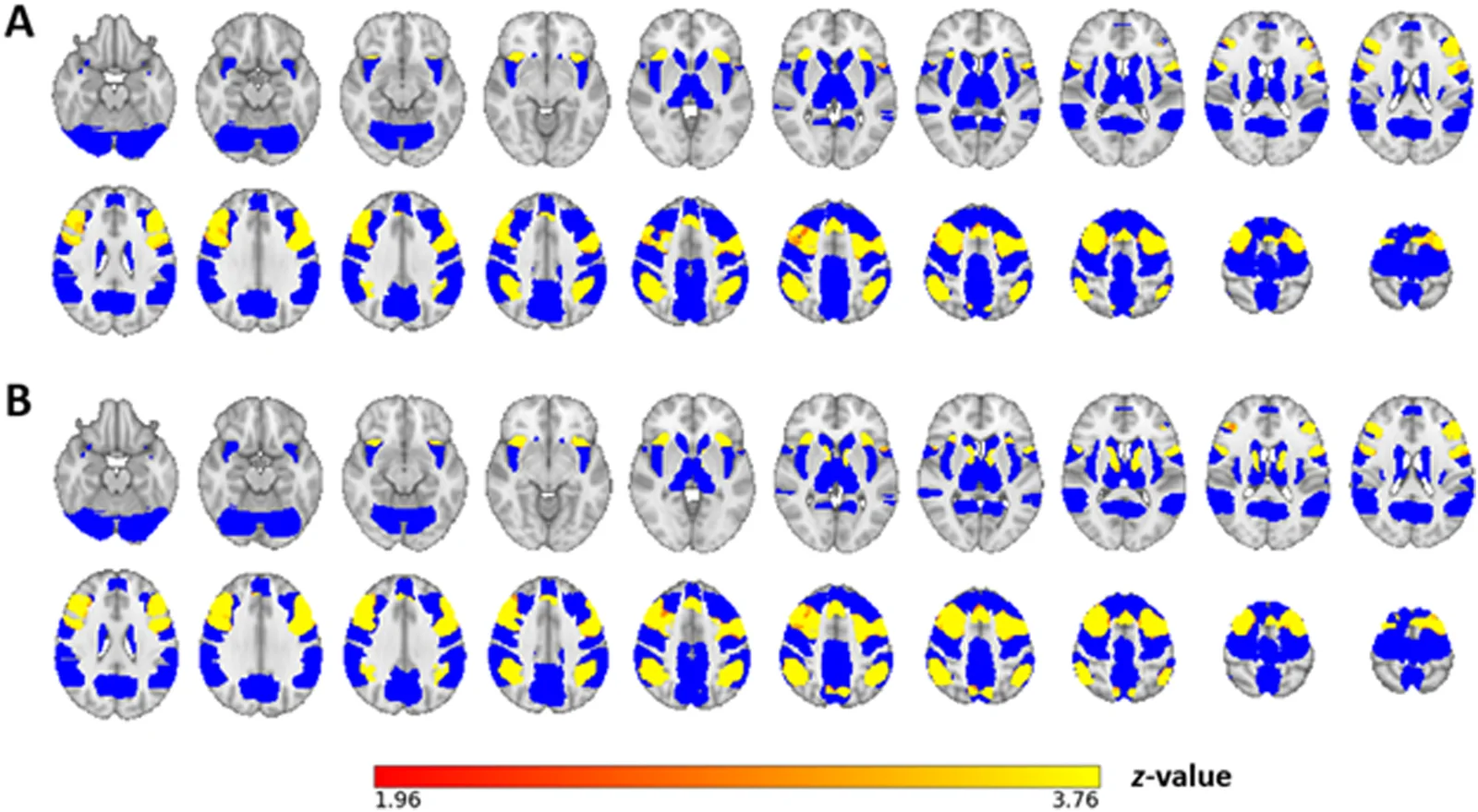
Functional activation during the n-back fMRI task within the working memory mask. (A) The following categorical contrast was used: 0-back versus 1- to 3-back conditions with weights [-3, 1, 1, 1]. (B) The following linear contrast was used: 0-back to 3-back conditions with weights [-1.5, −0.5, 0.5, 1.5]. The models were corrected for age and sex. Functional activation is shown in red-yellow, and the working-memory mask is shown in blue. Image is displayed in radiological orientation, spanning from Montreal Neurological Institute coordinates Z = −20 (top left) to Z = 66 (bottom-right). (For interpretation of the references to colour in this figure legend, the reader is referred to the Web version of this article.)

Working memory performance (d-prime, reaction time, and accuracy) did not relate to functional activation within the working memory mask or across the whole-brain, neither before nor after controlling for diagnostic status (*p* > 0.05). Additionally, none of the three immuno-metabolic measures were associated with working memory performance or with functional activation across diagnostic groups (*p* > 0.05). Standardized beta estimates of all associations between immuno-metabolic measures and working memory performance are provided in Supplemental Table 4. Finally, no significant interaction effects with diagnostic status were observed (*p* > 0.05).

The exploratory analyses revealed that the individual inflammatory markers were not related to working memory performance or working memory load-related functional activation across groups when applying Bonferroni correction to the four markers tested (*p* > 0.0125). However, without this correction, higher CRP and IL-6 were associated with decreased activation in the precuneus on the linear contrast (CRP Z-max: 3.54, x = 14, y = −56, z = 46 and IL-6: Z-max: 3.72, x = 14, y = −62, z = 18) (Supplemental Fig. 2), with IL-6 also showing a similar effect on the categorical contrast (Z-max: 3.72, x = 24, y = −56, z = 24) (all *p* uncorrected < 0.05). These exploratory findings should be considered hypothesis-generating. Additionally, the 0-back versus 2-back contrast elicited expected activation within large sections of the working memory mask (*p* < 0.05; Supplemental Fig. 3). This contrast revealed no significant associations between functional activation and immuno-metabolic markers (*p* > 0.05). However, higher d-prime scores in the 2-back condition related to lower functional activation within the right superior frontal gyrus before and after correcting for diagnostic status (Z-max = 3.72, x = 16, y = 34, z = 56, *p* < 0.05) (Supplemental Fig. 4).

## 4 Discussion

This study investigated relationships between immuno-metabolic markers, working memory performance, and functional brain activation in persons with depressive and anxiety disorders and healthy controls. While the n-back task robustly recruited the expected brain regions, we found no evidence that immuno-metabolic markers were related to either working memory performance or working memory load-related functional activation.

Our findings do not indicate that relations between immuno-metabolic markers and working memory performance are entirely absent, but instead add to the growing evidence that such relationships may be difficult to detect within a cross-sectional framework, likely due to the greater temporal stability of working memory performance compared with the more fluctuating immuno-metabolic markers (Morrens et al., 2022; Mac Giollabhui et al., 2025). Importantly, these findings do not exclude a role for central immuno-metabolic processes. Future research should incorporate more direct measures of central processes, such as positron emission tomography imaging of microglial inflammation (Meyer et al., 2020), diffusion MRI to assess inflammation-induced microstructural changes (Plank et al., 2025), or cerebrospinal fluid analysis of neuroinflammatory biomarkers (Sorensen et al., 2022). Additionally, genetic or epigenetic markers of chronic inflammation (Stevenson et al., 2020), which are less prone to state-related influences (e.g., recent infections) than peripheral inflammation markers, as well as other indicators of metabolic dysfunction, such as alterations in the kynurenine pathway (Pan et al., 2025), may provide complementary insights. Furthermore, we did observe a nominal association between higher CRP/IL-6 levels and reduced functional activation in the precuneus, which should be considered hypothesis-generating, and could inform future studies investigating such associations. Additionally, it remains plausible that chronic low-grade systemic inflammation and metabolic dysregulation contribute to cumulative alterations in brain function that only manifest in cognitive impairment after prolonged exposure. Indeed, prior studies investigating relations between inflammation and cognition have reported null findings in cross-sectional analyses but observed significant associations in longitudinal designs (Mac et al., 2024). Furthermore, a meta-analysis of interventional studies using immunomodulatory agents has demonstrated improvements in working memory performance in psychotic disorders (Jeppesen et al., 2020). Accordingly, longitudinal and interventional studies, in combination with multi-modal neuroimaging (fMRI, diffusion tensor imaging, positron emission tomography), will be crucial for clarifying the temporal and mechanistic links between immuno-metabolic dysregulation and cognitive impairment. For instance, future work could focus on investigating the impact of low-grade systemic inflammation and metabolic dysregulation across multiple time-points over several years to determine whether they predict cognitive impairment and progressive disruptions in brain function.

The observation that our n-back task elicited increased functional activation in large sections of the working memory mask aligns with prior studies (Wang et al., 2019; Yaple et al., 2019) and supports the validity of the experimental paradigm. However, although a large-scale meta-analysis (N = 1666) reported small-to-moderate deficits in accuracy and slower reaction times across 1-, 2-, and 3-back conditions in persons with depression (Nikolin et al., 2021), our primary analyses (N = 123) revealed no significant case-control differences. This discrepancy may be attributed to the high degree of cognitive heterogeneity within depressed cohorts, as suggested by the substantial between-study heterogeneity in the meta-analysis (Nikolin et al., 2021), or to limited statistical power to detect subtle differences in the current sample. Notably, we observed that higher symptom severity was associated with poorer working memory performance across groups, indicating that a dimensional approach may be more sensitive to cognitive differences than categorical diagnosis. This may partly reflect heterogeneity within and overlap across diagnostic categories, which can obscure associations that are better captured along a continuous dimension. This aligns with the Research Domain Criteria framework (Insel et al., 2010), which conceptualizes working memory as a transdiagnostic construct that varies along a continuum spanning normal functioning to clinical impairment, thereby relating more closely to symptom severity than diagnostic status. In addition, our exploratory analyses revealed that persons with depressive and anxiety disorders showed lower d-prime and accuracy scores in 1- and 2-back conditions and slower reaction times in the 1-back and 3-back conditions. It may be hypothesized that 3-back performance might not exclusively reflect cognitive capacity, but may also be confounded by psychological factors such as stress or reduced motivation under high cognitive load. Indeed, prior evidence indicates that achievable working memory tasks are more intrinsically motivating than complex n-back tasks (Ramme et al., 2022). Collectively, the task-related neural patterns and the working memory performance differences and their relationships with symptom severity observed in the exploratory analyses suggest that the absence of associations with immuno-metabolic markers is unlikely to be attributable to poor task sensitivity.

Additionally, while a prior large-scale study by Owens et al. (2018) reported associations between working memory performance and functional activation, we observed no such relationships in our primary analyses. Notably, even in that study (Owens et al., 2018), functional activation explained only a minority of variation in performance, suggesting that additional factors contribute to individual differences in task performance. In contrast to the positive correlations between accuracy and mean functional activation across the total right superior frontal gyrus reported by Owens et al. (2018), our exploratory analyses revealed a negative association between 2-back d-prime scores and activation in a specific cluster within this region. The superior frontal gyrus is functionally heterogeneous, comprising both task-positive components (i.e., central executive network) and task-negative components (i.e., default mode network) (Li et al., 2013). In our sample, this cluster showed no significant task-evoked activation, suggesting it does not function as part of the task-positive network. Consequently, the observed negative association between superior frontal gyrus activation and d-prime scores may reflect more efficient suppression of task-irrelevant activity in high-performing individuals, consistent with findings by Owens et al. (2018) linking task-negative network deactivation to better working memory performance. Finally, we observed no case-control differences in immuno-metabolic measures, which may be present only in specific psychiatric subpopulations (e.g., ∼30% of persons with depression (Penninx et al., 2025)). Given the adequate variability in these measures within our sample, this is unlikely to account for the null findings in working memory performance and functional brain activation.

This study is the first to integrate extensive immuno-metabolic profiling with behavioral and neuroimaging measures to examine how immuno-metabolic markers relate to working memory performance and its neural correlates in depressive and anxiety disorders, using preregistered analyses. Nevertheless, several limitations should be acknowledged. First, peripheral immuno-metabolic measures were used as proxies for neuroinflammation and brain metabolic function, though their link with central markers is not fully established (Felger et al., 2020; Gigase et al., 2023). Second, while we used the n-back task to assess cognitive function in relation to immuno-metabolic factors, it primarily measures working memory capacity. Consequently, the impact of these immuno-metabolic factors on other cognitive domains and their associated neural correlates in depression and anxiety remains to be further investigated. Finally, neurobiological subtypes of depressive and anxiety disorders may exist (Li et al., 2025), potentially obscuring immuno-metabolic associations with working memory and functional activation across the whole sample. Future studies with substantially larger samples stratifying by neurobiology, symptom severity, and inflammatory burden (e.g., CRP > 3) could help clarify whether associations exist within biologically-enriched psychiatric subpopulations.

## 5 Conclusion

This study found no evidence that peripheral immuno-metabolic markers are related to working memory performance and working memory related functional brain activation in depressive and anxiety disorders and healthy controls. Future studies should employ longitudinal or interventional designs and incorporate more direct measures of neuroinflammation to clarify whether immuno-metabolic processes contribute to cognitive impairment in these psychiatric populations.

## CRediT authorship contribution statement

**Simon Braak:** Writing – original draft, Visualization, Software, Methodology, Formal analysis, Conceptualization. **Laura K.M. Han:** Writing – review & editing, Supervision, Project administration, Methodology, Investigation, Data curation, Conceptualization. **Chris Vriend:** Writing – review & editing, Methodology, Conceptualization. **Thomaz F.S. Bastiaanssen:** Writing – review & editing, Supervision, Conceptualization. **Brenda W.J.H. Penninx:** Writing – review & editing, Supervision, Funding acquisition, Conceptualization.

## Ethics declaration

Written informed consent to take part in the study and to publish the article has been obtained from all participants or their legal representatives. The privacy rights of participants have been observed.

This study included organ or tissue donors. This study includes human biological material and consent was obtained by donors, or their next of kin or legal representatives, for use in this study and for publication of the article. The samples used in this research were not sourced from executed prisoners or prisoners of conscience.

This study was performed in compliance with relevant laws, regulatory frameworks and guidelines where the research took place. This study was approved by the VU University Medical Center Amsterdam. (Approval No. 12-064)

The results of this clinical trial and any associated work have been posted in a registry. This clinical trial was registered with number NTR3460 (Netherlands Trial Register).

## Funding

The POWER project received funding from 10.13039/501100001826ZonMw under the umbrella of the Partnership Fostering a European Research Area for Health (ERA4Health) (GA N° 101095426 of the EU Horizon Europe Research and Innovation Programme). The MOTAR study was funded by NWO-VICI grant (number 91811602) by Prof. Dr. B.W.J.H. Penninx.

## Declaration of competing interest

All authors declare that they have nothing to disclose.

## Data Availability

Data will be made available on request.
