# Supplementary material for: Associations between immuno-metabolic markers, working memory, and functional brain activation in depressive and anxiety disorders
Source: Brain Behav Immun Health. 2026 Sep 22;57:101369. doi: 10.1016/j.bbih.2026.101369 (PMC13625634; doi:10.1016/j.bbih.2026.101369)
Supplement: Supplementary file 1 [file mmc1.docx]

***Supplement Braak et al.,***

**2. Methods**

***MRI data acquisition***

T1-weighted structural MRI images were acquired with the following parameters: repetition time (TR) = 8.1 ms; echo time (TE) = 3.7 ms, flip angle 8°; field of view (FoV) = 240 x 240; and 1 mm isotropic voxel resolution. T2*-weighted gradient-echo echoplanar imaging scans were used to acquire task-based fMRI data with the following parameters: 230 whole-brain volumes, TR = 2300 ms; TE = 27.6 ms; flip angle 76 degrees; in-plane voxel size 3 x 3 x 3.3 mm, 37 sequentially ascending slices with 3 mm thickness and 0.3 mm slice gap; scan duration = ~ 9.5 minutes.

***Metabolic markers***

**Supplemental Table 1.** Metabolite loadings on principal component 1.

| **Metabolite** | **Unit** | **Group** | **Metabolite PC1 (42.3%)** |
| --- | --- | --- | --- |
| Total lipids in lipoprotein particles | mmol/l | Total lipids | 0.17 |
| VLDL cholesterol | mmol/l | Cholesterol | 0.17 |
| Concentration of VLDL particles | mmol/l | Lipoprotein particle concentrations | 0.17 |
| Free cholesterol in VLDL | mmol/l | Free cholesterol | 0.17 |
| Cholesteryl esters in VLDL | mmol/l | Cholesteryl esters | 0.17 |
| Remnant cholesterol | mmol/l | Cholesterol | 0.17 |
| Apolipoprotein B | g/l | Apolipoproteins | 0.17 |
| Polyunsaturated fatty acids | mmol/l | Fatty acids | 0.17 |
| Phospholipids in VLDL | mmol/l | Phospholipids | 0.17 |
| Concentration of LDL particles | mmol/l | Lipoprotein particle concentrations | 0.17 |
| Total fatty acids | mmol/l | Fatty acids | 0.17 |
| Non-HDL cholesterol | mmol/l | Cholesterol | 0.17 |
| Omega-6 fatty acids | mmol/l | Fatty acids | 0.17 |
| Cholesteryl esters in LDL | mmol/l | Free cholesterol | 0.16 |
| Total free cholesterol | mmol/l | Cholesteryl esters | 0.16 |
| Total lipids in LDL | mmol/l | Total lipids | 0.16 |
| Total lipids in VLDL | mmol/l | Total lipids | 0.16 |
| Linoleic acid | mmol/l | Fatty acids | 0.16 |
| Phospholipids in LDL | mmol/l | Phospholipids | 0.16 |
| LDL cholesterol | mmol/l | Cholesterol | 0.16 |
| Monounsaturated fatty acids | mmol/l | Fatty acids | 0.15 |
| Saturated fatty acids | mmol/l | Fatty acids | 0.15 |
| Total cholesterol | mmol/l | Cholesterol | 0.15 |
| Clinical LDL cholesterol | mmol/l | Cholesterol | 0.15 |
| Total triglycerides | mmol/l | Triglycerides | 0.14 |
| Total esterified cholesterol | mmol/l | Phospholipids | 0.14 |
| Total phospholipids in lipoprotein particles | mmol/l | Cholesteryl esters | 0.14 |
| Ratio of apolipoprotein B to apolipoprotein A1 | ratio | Apolipoproteins | 0.14 |
| VLDL triglycerides | mmol/l | Triglycerides | 0.14 |
| Free cholesterol in LDL | mmol/l | Triglycerides | 0.14 |
| LDL triglycerides | mmol/l | Free cholesterol | 0.14 |
| Phosphoglycerides | mmol/l | Other lipids | 0.12 |
| Total cholines | mmol/l | Other lipids | 0.12 |
| Ratio of triglycerides to phosphoglycerides | ratio | Other lipids | 0.12 |
| Phosphatidylcholines | mmol/l | Other lipids | 0.12 |
| Sphingomyelins | mmol/l | Other lipids | 0.11 |
| Omega-3 fatty acids | mmol/l | Fatty acids | 0.11 |
| Average diameter for VLDL particles | nm | Lipoprotein particle sizes | 0.11 |
| HDL triglycerides | mmol/l | Triglycerides | 0.11 |
| Valine | mmol/l | Amino acids | 0.10 |
| Glycoprotein acetyls | mmol/l | Inflammation | 0.10 |
| Total concentration of branched-chain amino acids | mmol/l | Amino acids | 0.10 |
| Leucine | mmol/l | Amino acids | 0.09 |
| Phenylalanine | mmol/l | Amino acids | 0.08 |
| Isoleucine | mmol/l | Amino acids | 0.08 |
| Lactate | mmol/l | Glycolysis related metabolites | 0.08 |
| Alanine | mmol/l | Amino acids | 0.07 |
| Glucose | mmol/l | Glycolysis related metabolites | 0.07 |
| Creatinine | μmol/l | Fluid balance | 0.06 |
| Total concentration of lipoprotein particles | mmol/l | Lipoprotein particle concentrations | 0.06 |
| Tyrosine | mmol/l | Amino acids | 0.05 |
| Histidine | mmol/l | Amino acids | 0.05 |
| Pyruvate | mmol/l | Glycolysis related metabolites | 0.04 |
| Docosahexaenoic acid | mmol/l | Fatty acids | 0.04 |
| Concentration of HDL particles | mmol/l | Lipoprotein particle concentrations | 0.02 |
| Citrate | mmol/l | Glycolysis related metabolites | 0.01 |
| Glutamine | mmol/l | Amino acids | 0.01 |
| Albumin | g/l | Fluid balance | 0.00 |
| Apolipoprotein A1 | g/l | Apolipoproteins | -0.01 |
| Free cholesterol in HDL | mmol/l | Free cholesterol | -0.01 |
| 3-Hydroxybutyrate | mmol/l | Ketone bodies | -0.01 |
| Acetoacetate | mmol/l | Ketone bodies | -0.02 |
| Phospholipids in HDL | mmol/l | Phospholipids | -0.02 |
| Total lipids in HDL | mmol/l | Total lipids | -0.02 |
| Average diameter for LDL particles | mmol/l | Amino acids | -0.02 |
| Glycine | nm | Lipoprotein particle sizes | -0.03 |
| Acetone | mmol/l | Ketone bodies | -0.03 |
| Degree of unsaturation | degree | Fatty acids | -0.03 |
| HDL cholesterol | mmol/l | Cholesterol | -0.05 |
| Acetate | mmol/l | Ketone bodies | -0.05 |
| Cholesteryl esters in HDL | mmol/l | Cholesteryl esters | -0.05 |
| Average diameter for HDL particles | nm | Lipoprotein particle sizes | -0.08 |

The percentage of metabolite principal component 1 (PC1) indicates the proportion of explained variance in metabolites in the principal component analysis. VLDL = very low-density lipoprotein. LDL = low-density lipoprotein. HDL = high-density lipoprotein.

**3. Results**

**Supplemental Table 2**. Pearson’s r correlations between inflammatory markers.

| **Inflammatory markers** | **Correlation** | p-value |
| --- | --- | --- |
| CRP - IL-6 | r = 0.47 | **p < 0.001** |
| CRP - TNF-α | r = 0.10 | p = 0.259 |
| CRP - IFN-γ | r = 0.15 | p = 0.090 |
| IL-6 - TNF-α | r = 0.32 | **p < 0.001** |
| IL-6 - IFN-γ | r = 0.21 | **p = 0.018** |
| TNF-α - IFN-γ | r = 0.40 | **p < 0.001** |

CRP = C-reactive protein. IL-6 = Interleukin-6. TNF-α = Tumor necrosis factor-alpha. IFN-γ = Interferon-gamma.

**Supplemental Table 3.** Working memory performance for each load condition.

|  | **Persons with depressive and anxiety disorders**  (N=54) | **Healthy controls**  (N=69) | Case-control differences |
| --- | --- | --- | --- |
| **0-back** |  |  |  |
| d-prime, mean (SD) | 3.9 (0.4) | 3.9 (0.3) | t(105.4) = -0.22, p = 0.830 |
| Reaction time, median (Q1 – Q3) | 405.4 (373.2–456.4) | 395.9 (359.4–423.5) | W = 2238.5, p = 0.056 |
| Accuracy (%), median (Q1 – Q3) | 1.0 (1.0–1.0) | 1.0 (1.0–1.0) | W = 1908, p = 0.698 |
| **1-back** |  |  |  |
| d-prime, mean (SD) | 3.8 (0.4) | 4.0 (0.3) | t(87.5) = -2.61, **p = 0.011** |
| Reaction time, median (Q1 – Q3) | 458.5 (414.2–536.4) | 434.0 (374.5–492.4)  ) | W = 2281, **p = 0.033** |
| Accuracy (%), median (Q1 – Q3) | 1.0 (0.9–1.0) | 1.0 (1.0–1.0) | W = 1492, **p = 0.014** |
| **2-back** |  |  |  |
| d-prime, mean (SD) | 3.2 (0.7) | 3.6 (0.5) | t(92.1) = -2.94, **p = 0.004** |
| Reaction time, median (Q1 – Q3) | 531.4 (467.3–656.7) | 507.0 (451.1–583.9) | W = 2087.5, p = 0.254 |
| Accuracy (%), median (Q1 – Q3) | 0.9 (0.8–1.0) | 0.9 (0.9–1.0) | W = 1357, **p = 0.008** |
| **3-back** |  |  |  |
| d-prime, mean (SD) | 2.7 (0.8) | 2.8 (0.6) | t(97.8) = -0.69, p = 0.493 |
| Reaction time, median (Q1 – Q3) | 599.3 (522.3–715.9) | 538.0 (459.9–609.2) | W = 2339, **p = 0.015** |
| Accuracy (%), median (Q1 – Q3) | 0.8 (0.6–0.9) | 0.8 (0.7–0.9) | W = 1802.5, p = 0.757 |

**Supplemental Table 4.** Standardized beta estimates of associations between immuno-metabolic markers and working memory performance.

| **Association** | **Standardized beta estimates with 95% CI** | p-value |
| --- | --- | --- |
| d-prime - Inflammation | -0.01 [-0.20, 0.17] | p = 0.880 |
| Reaction time - Inflammation | -0.04 [-0.22, 0.14] | p = 0.658 |
| Accuracy - Inflammation | -0.10 [-0.28, 0.09] | p = 0.302 |
| d-prime - Metabolic syndrome | -0.11 [-0.32, 0.11] | p = 0.328 |
| Reaction time - Metabolic syndrome | 0.03 [-0.17, 0.23] | p =0.777 |
| Accuracy - Metabolic syndrome | -0.18 [-0.39, 0.02] | p = 0.080 |
| d-prime - Metabolomics factor | -0.00 [-0.21, 0.20] | p = 0.964 |
| Reaction time - Metabolomics factor | 0.04 [-0.15, 0.23] | p = 0.693 |
| Accuracy - Metabolomics factor | -0.05 [-0.24, 0.15] | p = 0.626 |

All associations were corrected for age, sex, and diagnostic status.

**Supplemental Figure 1.** Functional activation during the n-back fMRI task across the whole-brain. (A) The following categorical contrast was used: 0-back versus 1- to 3-back conditions with weights [-3, 1, 1, 1]. (B) The following linear contrast was used: 0-back to 3-back conditions with weights [-1.5, -0.5, 0.5, 1.5]. The models were corrected for age and sex. Functional activation is shown in red-yellow. Image is displayed in radiological orientation, spanning from Montreal Neurological Institute coordinates Z = -20 (top left) to Z = 66 (bottom-right).

**Supplemental Figure 2.** Brain maps illustrating the relationship between higher (A) CRP and (B) IL-6 levels and lower functional activation within the precuneus. Results are thresholded at the uncorrected p-value and are shown for the linear contrast. Clusters are displayed in red-yellow and shown in radiological orientation. Peak Montreal Neurological Institute voxel coordinates are as follows: (A) x = 14, y = -56, z = 46; (B) x = 14, y = -62, z = 18. Associations were corrected for age, sex, and diagnostic status.

**Supplemental Figure 3.** Functional activation during the n-back fMRI task within the working-memory mask using the 0-back versus 2-back contrast. Functional activation is shown in red-yellow, and the working-memory mask is shown in blue. Image is displayed in radiological orientation, spanning from Montreal Neurological Institute coordinates Z = -20 (top left) to Z = 66 (bottom-right). The model was corrected for age and sex.

**Supplemental Figure 4.** Brain maps illustrating the relationship between higher d-prime scores in the 2-back condition and lower functional activation within the right superior frontal gyrus. Results are based on the 0-back versus 2-back contrast, with the association corrected for age, sex, and diagnostic status. The cluster is displayed in red-yellow and shown in radiological orientation, with peak Montreal Neurological Institute voxel coordinates x = 16, y = 34, z = 56.
